# Vacuum-assisted thrombectomy for intracardiac and intravascular thrombi: A single-center experience

**DOI:** 10.1016/j.xjse.2025.100068

**Published:** 2025-11-06

**Authors:** Adam M. Carroll, Kenaz Bakdash, Christian Ghincea, Nicolas Chanes, Kristofer Schramm, Tamas Seres, T. Brett Reece, Matthew Zipse, Robert Reyes, Leigh Casadaban, Paul J. Rochon, Muhammad Aftab

**Affiliations:** a Department of Surgery, University of Colorado, Aurora, Colo; b Department of Radiology, University of Colorado, Aurora, Colo; c Department of Anesthesiology, University of Colorado, Aurora, Colo; d Department of Cardiothoracic Surgery, University of Colorado, Aurora, Colo; e Department of Cardiology, University of Colorado, Aurora, Colo

**Keywords:** endovascular, intracardiac thrombus, intravascular thormbus, minimally invasive

## Abstract

**Objective:**

Percutaneous vacuum-assisted mechanical thrombectomy using venovenous bypass is a potential alternative to open surgery for the removal of intracardiac and intravascular/caval thrombi. We sought to evaluate the unique role and efficacy of this procedure in the treatment of high-risk patients deemed poor candidates for surgical thrombectomy.

**Methods:**

Between May 2015 and October 2023, 40 patients underwent vacuum-assisted thrombectomy for intracardiac or caval thrombi using venovenous bypass at our medical center. Patient and case characteristics, procedural details, and postprocedural outcomes were collected retrospectively. All procedures involved a multidisciplinary approach by cardiac surgeons, interventional radiologists, and cardiac anesthesiologists. Cardiac electrophysiologists also participated in the procedures when patients needed concomitant cardiovascular implantable electronic device system extractions.

**Results:**

Mean age of patients was 50 ± 16 years. Indications included indwelling catheter thrombus (n = 10; 25%), cardiovascular implantable electronic device infection (n = 12, 30%), tricuspid endocarditis (n = 8, 20%), and bland and tumor thrombus (n = 11, 28%, n = 7, 18%, respectively). Successful removal rate (>70% of thrombus removed) was 85% (n = 34). Interventional adjuncts included cardiovascular implantable electronic device lead extraction (n = 10, 25%) and snaring from contralateral access site (n = 7, 18%). In-hospital and 30-day mortality were 5% (n = 2) and 8% (n = 3), respectively. Complications included postoperative red blood cell transfusion (n = 5, 13%), pulmonary embolism (n = 2, 5%), and recurrent thrombosis (n = 2, 5%). Median total follow-up time was 16 ± 3 months, with either complete resolution or decreased burden of residual thrombus.

**Conclusions:**

Vacuum-assisted thrombectomy is a rapid, effective, and safe technique when treating critically ill patients with acute intracardiac and caval thrombi and vegetation. It can be a highly valuable adjunct when treating poor candidates for open cardiovascular surgery.

**Figure:**
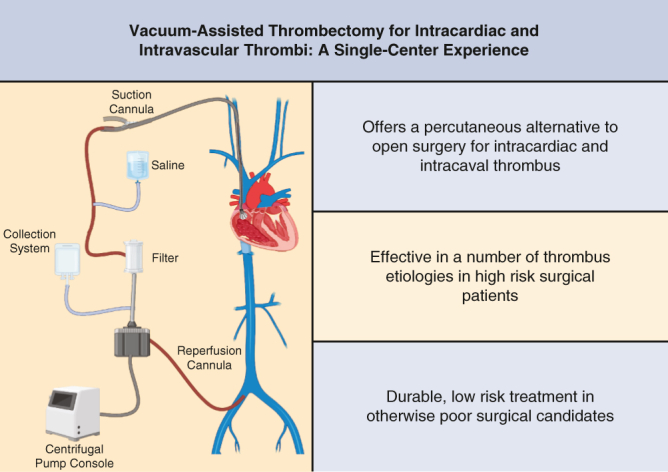
Vacuum thrombectomy cannulation circuit setup and procedural benefits.

**Central Message**

Percutaneous vacuum-assisted mechanical thrombectomy is a viable minimally invasive option to treat multiple etiologies of intracardiac and intracaval thrombus for poor surgical candidates.

**Perspective**

Intracardiac and intracaval thrombus pose a life-threatening risk, with high-risk surgical patients having limited procedural options that carry significant morbidity and mortality. This study found that percutaneous vacuum-assisted mechanical thrombectomy was effective for intracaval and intracardiac thrombus for a variety of etiologies and provided durable benefit extending 1 year post-thrombectomy.

Venous thromboembolism, encompassing deep vein thrombosis and pulmonary embolism (PE), affects 300,000 to 600,000 Americans annually.^1^ PE alone occurs in more than 250,000 individuals in the United States each year and is associated with sudden death in up to 25% of cases, contributing substantially to morbidity and mortality.^2^ Right heart thrombi are particularly dangerous due to their potential to cause PE and their association with hemodynamic instability, cardiac arrest, and increased mortality.^3^, ^4^, ^5^ The rising incidence of intravenous drug use—leading to infectious endocarditis—and the growing use of cardiac electronic devices—resulting in lead vegetations—have both contributed to the increased occurrence of right heart thrombi.^6^^,^^7^ Many patients with intracaval or intracardiac thrombi are not suitable candidates for thrombolytic therapy or open surgical thrombectomy, making percutaneous mechanical thrombectomy a viable alternative treatment option.

The AngioVac aspiration system (Angiodynamics) received Food and Drug Administration approval in 2009 for the removal of undesired intravascular thrombi or emboli using a venovenous extracorporeal bypass circuit. Divekar and colleagues^8^ were the first to report its successful use in extracting vegetations. Since that time, multiple studies have demonstrated its efficacy in removing intracaval and intracardiac thrombi.^9^, ^10^, ^11^ Additionally, off-label use of the AngioVac system in treating high-risk left heart thrombotic conditions has shown promising results, underscoring the need for more comprehensive outcome data.^12^, ^13^, ^14^

This study describes a large academic medical center's experience using this technique. We hypothesize that vacuum-assisted thrombectomy (VAT) using venovenous bypass is a rapid, effective, and safe technique in the treatment of high-risk patients with acute intracardiac and caval thrombi who are poor candidates for open cardiovascular surgery.

## Material and Methods

### Study Population

Between May 2015 and October 2023, 40 patients underwent VAT for intracardiac and caval thrombi at our institution. All procedures were performed in the hybrid operating room and involved a multidisciplinary team of cardiac surgeons, interventional radiologists, cardiac anesthesiologists, and cardiac electrophysiologists. Patient and case characteristics, procedural details, and postprocedural outcomes were collected retrospectively from electronic medical records. The Institutional Review Board at the University of Colorado approved the study (COMIRB#17-0198, February 6, 2017); individual informed consent was waived.

### Definitions

Thrombectomy success was defined as removal of more than 70% of thrombus by echocardiographic imaging. Bleeding was defined as any decrease in hemoglobin after the procedure and further differentiated as whether or not a transfusion is needed.

### Statistical Analysis

Results are presented as mean ± SD for quantitative variables and percentages (%) for categorical variables, except where indicated.

### AngioVac Thrombectomy Technique

The AngioVac device is a 22F l-vacuum cannula with funnel tip designed for en bloc removal of foreign materials such as thrombus, tumor, or vegetations from the vena cava, right heart, and pulmonary trunk via the use of extracorporeal bypass. By using a centrifugal pump console, the patient's blood is removed via the AngioVac cannula, passed through an external filter where the debris is captured, and returned via a reinfusion cannula into the patient's venous system.

All procedures are performed under general anesthesia. Fluoroscopy and intraoperative transesophageal echocardiography are used for guidance of the AngioVac cannula. For patients with conventional venous anatomy and nonocclusive thrombus in the inferior vena cava (IVC), bilateral common femoral vein access is obtained. A combination of either bilateral or unilateral internal jugular and common femoral venous access is used when thrombus is in a location that required a different approach for the AngioVac cannula.

By using the Seldinger technique, initial bilateral 5F vascular accesses are serially dilated to accommodate a 26F Dryseal sheath (Gore) on the AngioVac cannula side, and 16F on the side accepting the venous return cannula. Three vessel closure devices (Abbott) are placed in the 10, 12, and 2 o'clock positions around the AngioVac cannula sheath and at the 12 and 2 o'clock positions around the return cannula sheath. An intraprocedural arterial line is placed for hemodynamic monitoring. Heparin is given to maintain an activated clotting time of greater than 300 seconds throughout the procedure.

A 0.035-inch Lunderquist extra-stiff double-curved wire (Cook Medical) is used to achieve the delivery of the cannula to the desired location. The circuit is prime and de-aired, and the bypass circuit is then activated in a venovenous fashion. The centrifugal pump rate is set to 2 to 4 L/min. The AngioVac cannula is placed in close proximity to the clot or debris under fluoroscopic and transesophageal echocardiographic guidance and slowly advanced and redrawn. The aspirants are collected in the filter trap and later sent for microbiologic and cytologic analysis. Once the thrombus and the venous access devices are removed, the access sites are closed using a pre-close technique and protamine is administered to reverse the heparin. The patient is then monitored in the postanesthesia care unit.

## Results

We performed 40 procedures using VAT at our institution. Full patient characteristics are provided in Table 1. The average age of patients was 50 years. High-risk features were ubiquitous. These features included acute PE, prior sternotomy, current malignancy with active chemotherapy administration, septic shock, and a plethora of specific cardiovascular comorbidities including infective endocarditis, severe cardiomyopathy, chronic thromboembolic pulmonary hypertension, and concurrent extracorporeal membrane oxygenation (ECMO) use.

**Table 1:** Patient characteristics

Patient characteristics (n = 40)			
Patient comorbidities		High-risk features	
Age (y)	50 ± 16	Acute PE	19 (48%)
Male	20 (50%)	Acute respiratory failure	11 (28%)
Smoking	15 (38%)	Prior sternotomy	9 (23%)
Chronic lung disease	17 (43%)	Severe cardiomyopathy (EF <30)	6 (15%)
Diabetes	15 (38%)	Septic emboli	10 (25%)
Coagulopathy	16 (40%)	Septic shock	7 (18%)
Obesity	17 (43%)	Active chemotherapy	6 (15%)
Malignancy	12 (30%)	Pulmonary hypertension	8 (20%)
Chronic kidney disease (eGFR <60)	9 (23%)	Recent AKI	9 (23%)
Baseline creatinine	1.34 ± 1.21	Frailty	5 (10%)
Autoimmune disease	6 (15%)	Morbid obesity	7 (18%)
Immunosuppression	6 (15%)	CTEPH	3 (8%)
Polysubstance abuse	4 (10%)	Severe RV dysfunction	5 (13%)
Hepatitis C	2 (5%)	Bilateral lung transplantation	2 (5%)
Additional cardiovascular comorbidities		Cardiogenic shock	2 (5%)
Heart failure	17 (43%)	Concurrent ECMO	2 (5%)
Indwelling venous catheter	12 (30%)	Diabetic ketoacidosis	2 (5%)
Hypertension	18 (45%)	Chest wall radiation	1 (3%)
Prior cardiac surgery	10 (25%)	Cystic fibrosis	1 (3%)
Infective endocarditis	14 (35%)	Pregnancy	2 (5%)
Pacemaker	12 (30%)	Recurrent strokes	2 (5%)
Hyperlipidemia	9 (23%)		
Atrial fibrillation	7 (18%)		
Myocardial infarction	5 (13%)		
Coronary artery disease	4 (10%)		
Connective tissue disorder	2 (5%)		

Values are n (%) or mean ± SD. *EF*, Ejection fraction; *eGFR*, estimated glomerular filtration rate; *AKI*, acute kidney injury; *CTEPH*, chronic thromboembolic pulmonary hypertension; *ECMO*, extracorporeal membrane oxygenation.

The various indications for VAT included indwelling catheter thrombosis with and without superimposed infection, cardiovascular implantable electronic device infection (CIED) infection and vegetation, tricuspid endocarditis, bland thrombus, and tumor thrombus. Both intracardiac and exclusively extracardiac interventions were performed. Various procedural adjuncts were used to facilitate thrombus or CIED removal, or to treat the underlying cause of thrombosis. These included transvenous extraction of infected CIED leads and snaring of the early generation nonsteerable AngioVac catheters from the contralateral access site for directionality and concomitant use of handheld manual suction AlphaVac system (Angiodynamics). Details of the use of these operative adjuncts, full procedural details regarding indications, procedural characteristics, operative adjuncts, and access sites are described in Table 2.

**Table 2:** Procedural characteristics

Procedural indications & characteristics			
Procedure indication (n = 50)		Procedure characteristics (n = 28)	
Indwelling catheter thrombus	10 (20%)	Time from diagnosis to procedure (d)	18 ± 34
CIED infection and vegetation	12 (24%)	Procedure time (min)	186 ± 72
Tricuspid endocarditis	8 (16%)	EBL (mL)	132 ± 119
Bland thrombus	11 (22%)	AngioVac access sites	
Tumor thrombus	7 (14%)	RCF vein	25 (63%)
Indwelling catheter infection	2 (4%)	RIJ vein	15 (38%)
Thrombus location		LCF vein	3 (8%)
Intracardiac	35 (88%)	Venous return cannula sites	
RA	7 (20%)	LCF vein	28 (70%)
RA + RV + TV	6 (17%)	RCF vein	5 (13%)
RA + IVC	4 (11%)	LIJ vein	4 (10%)
RA + SVC	8 (23%)	RIJ vein	2 (5%)
RA + TV	2 (6%)	Operative adjuncts	
RA + PA	2 (6%)	Laser lead extraction	10 (25%)
RA + RV + TV + SVC	1 (3%)	Snaring from contralateral access site	7 (18%)
RA + RV + TV + IVC	5 (14%)	Indwelling catheter removal	6 (15%)
Extracardiac	5 (12%)	IVC filter removal	5 (13%)
IVC	4 (80%)	Rotational thrombectomy device	6 (15%)
PA + IVC	1 (20%)	Stent placement	2 (5%)
Thrombus length (cm)	3.7 ± 1.9	IVC venoplasty	2 (5%)
		Pulmonary embolectomy	1 (3%)
		ECMO	1 (3%)

Values are n (%) or mean ± SD. *CIED*, Cardiovascular implantable electronic device; *EBL*, estimated blood loss; *RCF*, right common femoral; *RIJ*, right internal jugular; *LCF*, left common femoral; *RA*, right atrium; *RV*, right ventricle; *TV*, tricuspid valve; *IVC*, inferior vena cava; *LIJ*, left internal jugular; *SVC*, superior vena cava; *PA*, pulmonary artery.

Procedural and postprocedural outcomes are provided in Table 3. Our success rate was 85%. Failure was encountered when attempting to remove chronic thrombi and early termination secondary to an intraprocedural pulmonary embolic event. Procedural and postprocedural complications were encountered in 8 patients and involved 5 instances of any in-hospital postoperative red blood cell transfusion, 2 perioperative pulmonary emboli, 2 instances of recurrent thrombosis, and 2 episodes of postprocedural pulseless electric activity (PEA) cardiac arrest. None of the patients experienced postprocedural bleeding, death, new postoperative bacteremia, or conversion to open surgical embolectomy.

**Table 3:** Procedural and postprocedural outcomes

Procedural and post-procedural outcomes	
Successful removal	34 (85%)
Average length of thrombus removed (cm)	3.6 ± 1.2
Complications	
Postoperative RBC transfusion	5 (13%)
PE	2 (5%)
Recurrent thrombosis	2 (5%)
PEA arrest	2 (5%)
Significant postoperative bleeding	0 (0%)
New postoperative bacteremia	0 (0%)
Mortality	9 (23%)
30-d mortality	3 (8%)
>30-d mortality	6 (15%)
In-hospital mortality	2 (5%)
Immediate postprocedure TEE (for intracardiac thrombus)	35 (100%)
Postprocedure TTE (for intracardiac thrombus)	33 (94%)
Average time to postprocedure echocardiography (d)	15 ± 32
Resolved	16 (48%)
Persistent thrombus	17 (52%)
Decreased	14 (82%)
Unchanged	2 (12%)
Increased	1 (6%)
Length of stay (d)	14 ± 14
Time to discharge after procedure (d)	9 ± 10
Follow-up	
Postdischarge follow-up	29 (73%)
Time from discharge to follow-up (d)	14 ± 13
Additional postdischarge TTE	16 (48%)
Average time from initial postprocedural TTE (mo)	46 ± 30
Resolved	11 (69%)
Decreased	5 (31%)
Median total follow-up time (mo)	16 ± 3

Values are n (%) or mean ± SD unless otherwise indicated. *RBC*, Red blood cell; *PE*, pulmonary embolism; *PEA*, pulseless electric activity; *TEE*, transesophageal echocardiography; *TTE*, transthoracic echocardiography.

The overall mortality during the follow-up was 23% with 30-day and in-hospital mortality rates of 8% and 5%, respectively. No procedural mortality occurred. There was an average of 9 days until discharge after the procedure.

Immediate postprocedure transesophageal echocardiography was performed on all patients treated for intracardiac thrombus. Further in-hospital and postdischarge transthoracic echocardiography (TTE) was performed on 33 of these patients with an average time to TTE of 15 days. Sixteen patients had no residual thrombus or vegetation, whereas 14 patients who demonstrated residual thrombus on initial TTE had significantly decreased thrombus burden. Two patients had an unchanged thrombus burden, and 1 patient had an increased thrombus burden. Sixteen patients who demonstrated persistent residual thrombus on the initial TTE had an additional TTE performed, with an average time between studies of 46 days. Of these, 11 patients had complete resolution of thrombus and 5 patients demonstrated decreased thrombus burden.

Twenty-nine patients received follow-up postdischarge. Initial follow-up occurred at an average of 14 days postdischarge. The patients were evaluated at 3 months, 6 months, and 12 months after the procedure with repeat TTE and chest computed tomography angiography. The median total follow-up time was 16 months.

## Discussion

Over the past decade, VAT has emerged as a viable alternative or adjunct to open embolectomy. A growing body of case reports, case series, and a 2019 meta-analysis has documented the use of the AngioVac device in similar scenarios.^8^^,^^9^^,^^15^, ^16^, ^17^, ^18^, ^19^, ^20^, ^21^, ^22^, ^23^ Since 2015, our institution has used AngioVac within an interdisciplinary team—comprising cardiac surgeons, interventional radiologists, cardiac anesthesiologists, and electrophysiologists—to manage a diverse range of intracaval and intracardiac thrombi in high-risk patients deemed unsuitable for open thrombectomy. In this study, we aim to share our institutional experience, highlighting the operative adjuncts used to optimize thrombus removal and address concomitant stenoses or emboli. We also provide our perspective on when AngioVac is most and least effective. Postoperative follow-up with serial TTE and computed tomography angiography was performed to evaluate early to midterm outcomes in this complex patient population.

All patients in our series exhibited multiple high-risk features that rendered them poor candidates for open surgical thrombectomy. Several presented with bland thrombi complicated by acute PE, which significantly increased their risk of right heart failure or death compared with those without PE.^24^ Additionally, many patients with infected CIED leads or indwelling catheters presented in septic shock, often accompanied by septic emboli and tricuspid valve vegetations.

Our series highlights numerous cases marked by complex pathology and high-risk comorbidities. One notable case involved successful removal of a 3.5-cm catheter-associated right atrial thrombus in a patient undergoing treatment for chronic myelocytic leukemia during an 18-week twin pregnancy. In another case, a patient on venovenous ECMO after bilateral lung transplantation developed extensive IVC thrombosis extending into the ECMO cannula. After thrombus removal, the ECMO catheter was cleared, and the patient was successfully decannulated. Several indwelling catheters and infected CIEDs were also extracted, including a particularly challenging case involving an infected biventricular automatic implantable cardioverter-defibrillator with abandoned pacing leads and vegetations extending from the superior vena cava (SVC) to the right ventricle. This patient, who had severe cardiomyopathy due to sarcoidosis, had complete heart block with 100% pacemaker dependence, and was immunosuppressed, presented in septic shock and underwent successful device removal.

Multiple procedural adjuncts were used to facilitate successful removal of thrombi and vegetations. All procedures were completed within the 6-hour limit recommended by AngioDynamics. In all cases involving infected CIEDs, transvenous lead extraction was performed by a collaborative team of cardiac surgeons and electrophysiologists. Rusia and colleagues^19^ reported a 97.7% success rate with this technique, and we achieved a 100% success rate in our series. Briefly, after AngioVac-assisted debulking of vegetations and thrombus from the infected leads—under combined fluoroscopic and transesophageal echocardiographic guidance—laser or TightRail sheaths (Spectranetics) were advanced over the leads to facilitate extraction. A Spectranetics Bridge occlusion balloon was pre-positioned via a wire placed in either the right internal jugular vein or left-sided access to provide rapid control in the event of SVC or subclavian vein injury.

During indwelling catheter and CIED removal, the AngioVac cannula was positioned near the SVC to capture any fibrin sheaths or thrombi dislodged during extraction. This technique was associated with no instances of acute PE in our cohort. In earlier cases, before the availability of a steerable AngioVac cannula, we used contralateral venous access for snaring to improve cannula positioning. A 15-mm gooseneck snare was typically introduced through an 8F sheath in the left common femoral vein and used to engage the leading edge of the AngioVac cannula, enhancing maneuverability into the right atrium, especially for difficult to access thrombi. This approach was particularly valuable before the introduction of the third-generation AngioVac cannula, which features 20° and 180° angulation for improved directional control. Additionally, snares were occasionally used to assist with CIED lead removal by directly grasping the leads.

A variety of adjunct techniques were used when managing acute and chronic thrombi within the SVC and IVC. One such adjunct was the AlphaVac cannula, which permits manual aspiration with greater control over pressure and volume. In 4 cases, the AlphaVac was used in tandem with the AngioVac device after the establishment of additional venous access. In these instances, the AngioVac provided both continuous aspiration and embolic protection during manual clot disruption via the AlphaVac. Additional techniques included mechanical balloon venoplasty and rotational thrombectomy using the Cleaner XT device (Argon Medical Devices). It has been proposed that in cases of chronic or high-burden thrombus, VAT alone may be insufficient due to limited flow rates.^25^ In 5 cases, IVC filter retrieval was necessary. Two patients required stenting of the IVC and iliac veins after combined vacuum-assisted and mechanical thrombectomy for acute-on-chronic thrombus in the setting of underlying venous stenosis.

Given the technical complexity and high medical acuity within our cohort, we consider our overall 85% success rate to be satisfactory. This outcome aligns with success rates reported by Basman and colleagues^26^ and Haupt and colleagues.^23^ Although some studies have reported higher success rates, these often included partial removals within their definition of success^21^ or were limited to a single indication, such as CIED-related infections or vegetations.^11^^,^^27^, ^28^, ^29^ Many of our successful cases shared characteristics previously associated with favorable outcomes, as described by D'Ayala and colleagues,^17^ including thrombus location in the caval system or right atrium, or clot-in-transit presentations. Thrombi and vegetations associated with CIEDs and indwelling catheters were particularly amenable to VAT in our experience.

Most of our technical failures occurred during attempts to aspirate chronic thrombi, which were often too firm and adherent to the vessel wall for successful removal. We achieved a 71% success rate in cases targeting tumor thrombi. One procedure was aborted due to an acute pulmonary embolic event while attempting to debulk a large IVC tumor thrombus in a patient with a known saddle embolus and metastatic embryonal carcinoma. Pathologic analysis of the partially removed thrombus confirmed tumor thrombus. This case highlights the need for caution when managing suspected tumor thrombi, particularly when the underlying etiology may not be evident preoperatively. In contrast, we successfully removed tumor thrombus in a patient with renal cell carcinoma, where the thrombus had a softer consistency, making it more amenable to vacuum-assisted extraction.

Patients with intracardiac or intracaval thrombi, particularly those with acute PE or tricuspid valve endocarditis, are known to have high overall mortality during follow-up.^5^^,^^30^ Our 30-day and in-hospital mortality rates were comparable to those reported in similar case series,^27^^,^^30^ and none of the observed deaths were due to the procedure itself. One patient with a large IVC thrombus extending into the ECMO cannula died of complications from bilateral lung transplantation 24 days postprocedure. Another patient, admitted with septic shock and tricuspid valve endocarditis related to an infected CIED, died despite a technically successful procedure, largely due to significant underlying comorbidities and progressive septic shock. For the remaining patients who died, death occurred at an average of 3.7 months after discharge, with mortality due to complications of their primary disease processes.

No intraoperative blood transfusions were required. Four units of packed red blood cells were administered postoperatively to 4 separate patients, all within 1 to 3 days of the procedure, each with preexisting chronic anemia. There was no evidence of active bleeding or access site complications. Our transfusion rate is at the lower end of those reported in the literature.^15^^,^^25^^,^^29^ This may reflect reduced intraoperative blood loss related to the AngioVac filtration mechanism.

One patient experienced PEA arrest 14 days after CIED extraction, due to prolonged bacteremia and underlying comorbidities rather than the procedure itself. Another patient had a PEA arrest on the day of a CIED replacement, 1 week after CIED extraction and AngioVac use; this was due to a vagal response and resolved with prompt hemodynamic recovery.

Only a few case series have published data regarding postdischarge follow-up for patients undergoing VAT.^15^^,^^28^^,^^29^ Average follow-up time over 16 months demonstrated 69% of intracardiac thrombi in our cohort had complete resolution of thrombus over that period. An additional 31% demonstrated decrease in size. We did lose 6 patients to follow-up from the intracardiac thrombi cohort, either due to patient death, noncompliance with scheduled appointments, or transfer to another hospital system. Patients with exclusively intracaval thrombus did not receive dedicated follow-up imaging.

### Limitations

Our study was limited by a small sample size and by its retrospective nature. Additionally, because of our attempts at providing a comprehensive review of a large variety of cases and indications, a detailed analysis of the role of the AngioVac device in specific situations, such as in the treatment of only tricuspid valve vegetations or treatment of only infected CIEDs, was not performed. However, future research from our database analyzing these specific subsets of indications is possible.

## Conclusions

**Figure 1:**
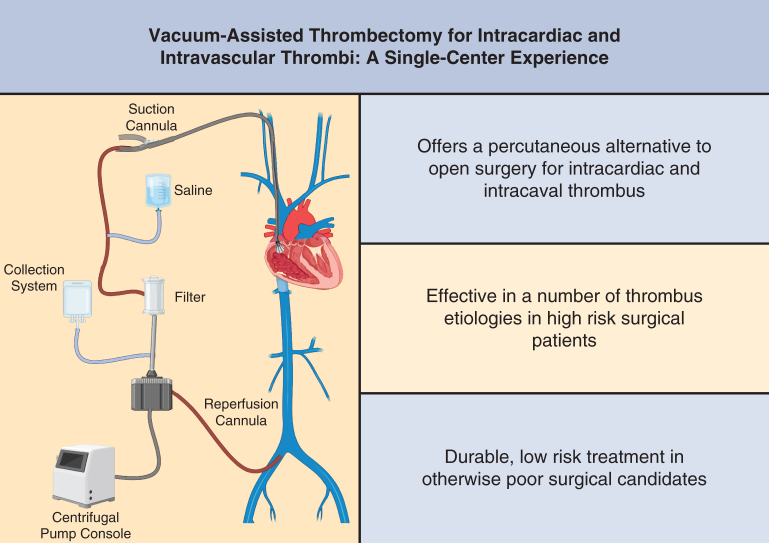
Central picture.

VAT using the AngioVac device has proven to be an effective option for patients with high surgical risk with intracaval or intracardiac thrombi from a variety of underlying etiologies. Furthermore, given the success of VAT demonstrated in this case series and the initial success in treating left heart pathology, the role of VAT in high-risk surgical candidates may continue to grow. When performed by an experienced, interdisciplinary team with strong technical proficiency, the procedure can achieve high success rates with low procedure-related morbidity (Figure 1).

## Conflict of Interest Statement

The authors reported no conflicts of interest.

The *Journal* policy requires editors and reviewers to disclose conflicts of interest and to decline handling or reviewing manuscripts for which they may have a conflict of interest. The editors and reviewers of this article have no conflicts of interest.
